# Evidence for Two Numerical Systems That Are Similar in Humans and Guppies

**DOI:** 10.1371/journal.pone.0031923

**Published:** 2012-02-15

**Authors:** Christian Agrillo, Laura Piffer, Angelo Bisazza, Brian Butterworth

**Affiliations:** 1 Department of General Psychology, University of Padova, Padova, Italy; 2 Institute of Cognitive Neuroscience, University College of London, London, United Kingdom; Georgia State University, United States of America

## Abstract

**Background:**

Humans and non-human animals share an approximate non-verbal system for representing and comparing numerosities that has no upper limit and for which accuracy is dependent on the numerical ratio. Current evidence indicates that the mechanism for keeping track of individual objects can also be used for numerical purposes; if so, its accuracy will be independent of numerical ratio, but its capacity is limited to the number of items that can be tracked, about four. There is, however, growing controversy as to whether two separate number systems are present in other vertebrate species.

**Methodology/Principal Findings:**

In this study, we compared the ability of undergraduate students and guppies to discriminate the same numerical ratios, both within and beyond the small number range. In both students and fish the performance was ratio-independent for the numbers 1–4, while it steadily increased with numerical distance when larger numbers were presented.

**Conclusions/Significance:**

Our results suggest that two distinct systems underlie quantity discrimination in both humans and fish, implying that the building blocks of uniquely human mathematical abilities may be evolutionarily ancient, dating back to before the divergence of bony fish and tetrapod lineages.

## Introduction

Numerousness, like shape, size and color, is a basic property of our perceptual world. It has long been recognized that adults, infants and non-human animals can instantly extract the numerical information from a visual scene without counting the elements [1], [2], [3], [4]. In humans, this ability is thought to be based on two distinct non-verbal systems that operate over different parts of the number range [5], [6], [7]. One is a system for representing approximate numerosities as analog magnitudes, and is usually referred to as the ‘analog magnitude system’ (ANS) [8], [9], [10] that has virtually no upper limit but is subject to a ratio limit in accordance with Weber's Law, which states that the capacity to discriminate between two quantities becomes increasingly accurate as the ratio between them increases. The second system is referred to as the ‘object-file system’ and is held to depend on a mechanism for representing and tracking small numbers of individual objects [3], [7], [11]. If this object-tracking system operates by keeping track of individual elements, it is precise but allows for the parallel representation of a small number of objects (usually three-four elements in adults). It is often assumed that this is the system that supports ‘subitizing’ – the accurate reporting of the numerosity of small sets without serial counting [6], [7]. The two mechanisms appear to differ in many respects, including speed, accuracy and cognitive load [11], [12].

The lack of a ratio effect is the main signature that allows experimental differentiation of the object-file system from the analog magnitude system [3]: the performance of an adult is very similar when discriminating 3 vs. 4 or 1 vs. 4 objects, whereas we are much more accurate in discriminating 5 from 20 objects than 15 from 20 objects. Not all the studies, however, reported a different ratio-effect between small and large numbers [13], [14], [15]. For instance, in a task requiring to apply an ordinal numerical rule, Cantlon and Brannon [16] also found evidence of a ratio-effect in the small number range. Recent neuropsychological, electrophysiological and brain imaging data suggest that these two non-verbal numerical systems probably have distinct neural substrates [17], [18], [19], [20], [21].

The analog magnitude system appears to be shared among many vertebrates. When required to determine the larger of two sets of elements, infants, macaques, dogs, swordtails and mosquitofish give approximate responses and their capacity to discriminate is strongly influenced by the numerical ratio [4], [16], [22], [23], [24]. Some have proposed that human infants and non-human vertebrates may also share with adults a distinct mechanism for precisely representing quantities up to four. However, evidence of separate systems in human infants and animals is less clear. In some studies, infants were found to discriminate different ratios in the small and large number range. For instance they were found to discriminate 2 vs. 3 but not 4 vs. 6 items in a habituation task despite the identical ratio difference [25]. Moreover, a study showed that children with Williams syndrome report a specific impairment in large number discrimination while the discrimination capacity in the small number range was unaffected [18]. Yet, vanMarle and Wynn [26] found that in infants the discrimination of auditory events was ratio-dependent also in the small number range, suggesting that infants use a single system of analog magnitude in the auditory domain.

Regarding non-human primates, chimpanzees have been shown to increase their reaction times to estimate the number of dots in a large number range but not in a small number range [27], but another study combining the data from four great apes found that numerical ratio was the best performance predictor in the range 0–6 [28]. In support of two-system hypothesis, rhesus monkeys successfully selected the greater group of apple slices with comparison of 1 vs. 2, 2 vs. 3 and 3 vs. 4 [29] while they discriminated between 4 and 8 lemons (1∶2 numerical ratio) but not between 4 and 6 (2∶3). However two recent studies reported that in rhesus monkeys accuracy was strongly affected by numerical ratio for both small and large quantities, in agreement with the existence of a single non-verbal mechanism over the whole numerical range [16], [30].

Several lines of evidence suggest that separate systems for small and large numbers may exist in other vertebrates too. In a recent work on attack/retreat decisions in free-ranging dogs [31], Bonanni and collaborators reported that dogs spontaneously assessed large quantities as noisy magnitudes. In contrast dogs approached aggressively with the same probability when they outnumbered opponents by a 1∶2, 2∶3 or 3∶4 ratio, suggesting that dogs discriminate two small quantities using an object-file mechanism. Among birds, New Zealand robins can discriminate 1 vs. 2, 2 vs. 3 and 3 vs. 4 but not 4 vs. 5 and 5 vs. 6; however these birds can also distinguish between large numbers provided that the numerical ratio is at least 1∶2 [32]. Studies on domestic chicks and bees have demonstrated that both species can successfully discriminate 2 vs. 3 while they fail to distinguish the same numerical ratio when two large numbers are presented, such as 4 vs. 6 [33], [34]. However, this kind of evidence for two numerical systems may not be conclusive. In some cases there are alternative explanations. For example, if chicks and bees can only represent small numbers, then they will not be able to discriminate 4 from 6.

In recent years, numerical competence has been investigated in several teleost species using either operant conditioning or spontaneous preferences [22], [23], [35], [36], [37], [38]. Two of these studies imply the possibility that separate systems for large and small numbers may exist even in fish. Mosquitofish have been found to discriminate between shoals differing in numerosity when the paired numbers were 1 vs. 2, 2 vs. 3 and 3 vs. 4 but they succeeded with only up to a 1∶2 numerical ratio (4 vs. 8 or 8 vs. 16) when they had to discriminate between large numbers [22]. In the second study it has been shown that one-day old guppies can discriminate between small quantities of social companions (<4), showing an inborn ability to elaborate small quantities, while the capacity to discriminate large quantities (4 vs. 8) emerges later, as a consequence of both maturation and social experience. This developmental dissociation indirectly suggests the existence of different systems for small and large quantities also in guppies [37].

Overall, the existence of a phylogenetically shared analog magnitude system appears generally accepted, but authors disagree as to whether a single analog magnitude mechanism accounts for discrimination over the whole numerical range, or a distinct system operates over the small number range.

In the present study, we used guppies to test one of the predictions of the hypothesis of two separate systems; according to this hypothesis fishes' ability to discriminate two large numbers should become more accurate as the difference between them increases while, in discriminating quantities <4, the performance should not be affected by the numerical ratio.

Guppies were required to choose the more numerous of two available groups of conspecifics. As reference, a group of undergraduate students were required to estimate the larger of two groups of dots while prevented from verbal counting. Both species were presented with the same five numerical ratios (0.25/0.33/0.50/0.67/0.75) both within the small number range (1–4) and beyond it. Furthermore, we examined the possible role of learning on performance of the fish, comparing subjects with or without previous experience of social groups.

## Materials and Methods

### Ethics Statement

The Experiment involving fish complies with all laws of the country (Italy) in which it was performed (D.M. 116192) and was approved by ‘Ministero della Salute’ (permit number: 6726-2011). The experiment with undergraduates was approved by the ethics committee of the Department of General Psychology of University of Padova and was conducted according to the Declaration of Helsinki. Before testing, all participants gave their written consent.

### Undergraduate experiment

In this experiment we adopted a procedure commonly used to measure non-verbal numerical abilities in adults, namely a computerized numerical judgement with sequential presentation of the stimuli [17], [36], [39]. Undergraduates were required to estimate the larger of two groups of dots while being prevented from using verbal counting.

#### Participants

A total of 18 undergraduate students (three males) between the ages of 18 and 31 (mean age: 21.33) took part in the present study for course credits. The experiments were carried out at the Department of General Psychology, University of Padova. All participants had normal or corrected vision.

#### Stimuli and procedure

The stimuli consisted of 240 pairs of arrays composed of different numbers of black dots. The dots differed in size and appeared in the center of the screen on a white background. The number of dots presented on the screen ranged from 1 to 24. Half of the pairs were controlled for continuous variables (cumulative surface area, density, luminance and overall space occupied by arrays), while the other half were not. Twenty further pairs, with identical features, were created for the initial training phase. The stimuli were displayed on a 17-inch monitor, using E-Prime software, in a darkened room.

**Figure 1:**
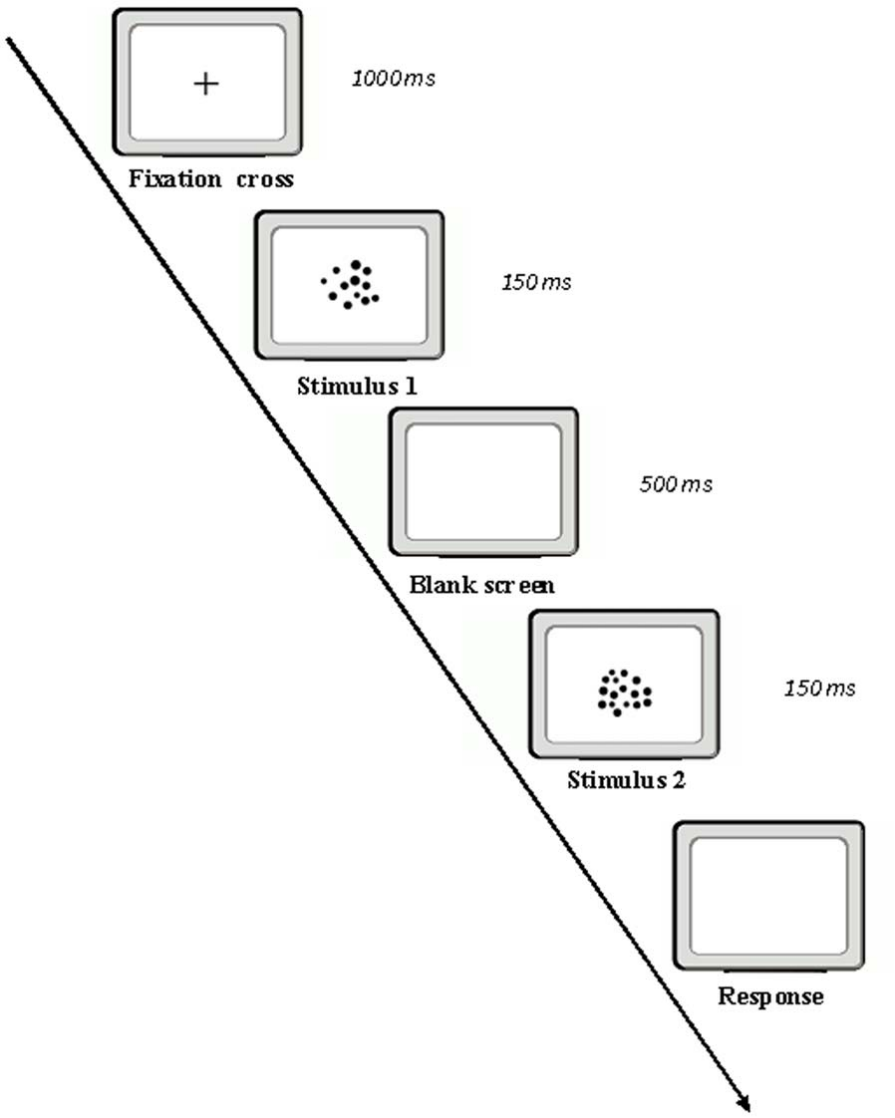
The experimental design used in the undergraduate experiment. The participants were sequentially presented with two groups of dots and had to estimate which group was more numerous.

After a period of dark adaptation, a short familiarization training phase with feedback was presented. The participants initially read the experimental instructions on the screen. A fixation cross then appeared in the center of the screen for 1000 ms, then a group of dots was displayed in the centre of the screen for 150 ms (Fig. 1). Following a 500 ms delay, the participants were shown another group of dots for 150 ms. They were required to estimate which one of the two groups was more numerous by pressing one of two keys on the keyboard. In half of the stimuli the larger group was presented first, in the other half the smaller group was presented first. They were instructed to make their responses as quickly and as accurately as possible. Furthermore, to prevent verbal processing of the stimuli, verbal suppression was introduced during the test by asking the participants to continuously repeat ‘abc’. No feedback was provided during the test.

Five numerical ratios were presented (0.25, 0.33, 0.50, 0.67, 0.75) for small (1 vs. 4, 1 vs. 3, 1 vs. 2, 2 vs. 3 and 3 vs. 4) and large (6 vs. 24, 6 vs. 18, 6 vs. 12, 6 vs. 9, 6 vs. 8) numerical contrasts. Reaction time is the common measure of numerical acuity in human studies, especially when, as in our study, participants must perform easy discriminations [17], [40], [41]. In these cases, in fact, participants commit few errors and variation in accuracy is potentially obscured by a ceiling effect. However since accuracy (proportion of time spent near the larger shoal) was the only variable measured in the fish experiment, for comparison, we also analyzed accuracy in the human experiment.

### Fish experiment

Like many other social fish, single guppies placed in an unknown environment show a strong tendency to join social companions and, if choosing between two shoals, they exhibit a preference for the larger one [37], [42]. This spontaneous tendency was used in this experiment to measure the ability of guppies to discriminate between two numerosities. During their life guppies might have different opportunity of familiarizing with large or small shoals and this could potentially affect the experiment. Because of this, alongside a sample of 140 adults, we tested a sample of 200 immature fish reared in pairs and therefore with no previous experience of social groups.

#### Subjects

The experienced subjects were adult females because they are more gregarious than males. They were reared in groups of 15 or more individuals. Seventy fish (14 in each numerical contrast) were tested in small quantity discriminations, and 70 were tested in large quantity discriminations. The inexperienced subjects were juvenile fish tested at the onset of their numerical abilities. A recent study showed that one-day-old guppies could discriminate the larger shoal when the choice was between numbers in the small number range, whereas the ability to discriminate large quantities appeared later, at approximately day 40 [37]. Thus, 100 one-day-old fish (20 in each numerical contrast) were tested in small quantity discrimination tasks, and 100 40-day-old fish were tested in large quantity discrimination tasks.

#### Stimuli and procedure

**Figure 2:**
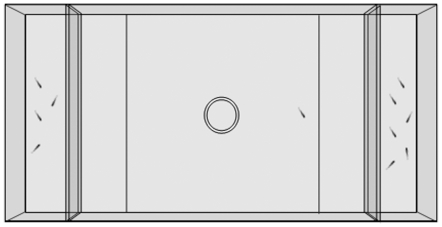
The experimental apparatus used in the fish experiment. Fish were individually placed into the middle of the apparatus where two shoals containing different numbers of fish were visible at the ends.

The experimental apparatus was composed of three adjacent tanks (Fig. 2). The central one, the ‘subject tank’, housed the test fish (36×60×35 cm). At the two ends two ‘stimulus tanks’ (36×10×10 cm) faced the subject tank. The apparatus was filled with 10 cm of water. The apparatus for the juvenile fish was similar to that used for the adult fish but reduced in size. The central ‘subject tank’ was 20×17.5×25 cm and the two ‘stimulus tanks’ were semi-octagonal shaped, with a side of 6.3 cm. This apparatus was filled with 4 cm of water and was partially modified when testing the 40-day-old fish by enlarging the stimulus tanks (semi-octagonal sides 8.3 cm long) and increasing the level of water (6 cm).

Two shoals containing different numbers of fish were placed into the stimulus tanks. The subjects were individually introduced into a transparent plastic cylinder (10 cm in diameter for the adults, and 3.5 cm for the juveniles) in the middle of the subject tank and allowed to acclimatize for two minutes. After this period the subject was observed for 15 minutes. Shoal preference was calculated as the time spent by the subject within a distance of 11 cm (4 cm when testing juveniles) from the glass facing either of the stimulus tanks. Subjects that did not visit either stimulus sector at least three times or spent less than 50% of the time in a choice area were considered inactive; they were discarded and replaced by another fish.

The same five numerical ratios of the students' experiment were presented to fish both for small (1 vs. 4, 1 vs. 3, 1 vs. 2, 2 vs. 3 and 3 vs. 4) and large (4 vs. 16, 4 vs. 12, 4 vs. 8, 4 vs. 6 and 6 vs. 8) numerical contrasts.

At the end of these trials, we increased the sample size for ratio 0.67 by testing 64 additional adult females, 32 in a small quantity discrimination (2 vs. 3) and 32 in a large quantity discrimination (4 vs. 6).

## Results

### Undergraduate experiment

#### Small numbers

**Figure 3:**
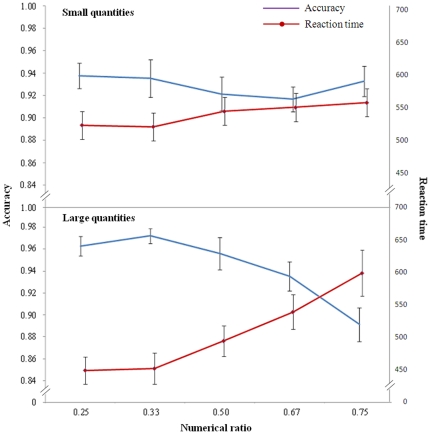
The results of the undergraduate experiment. Accuracy (proportion of correct responses) and reaction time are plotted against the numerical ratio of the contrasts for both large and small number (1–4) ranges. The performance of the participants showed ratio sensitivity for large numbers and ratio insensitivity in the small number range. Bars represent the standard error.

In accordance with the theory, reaction time was not affected by numerical ratio (ANOVA *F*_(4,68)_ = 1.474, *P* = 0.220, Fig. 3). Control of continuous variables did not affect performances (*F*_(1,17)_ = 0.421, *P* = 0.525; interaction *F*_(4,68)_ = 0.822, *P* = 0.516). To test if there was a significant slope, we performed a polynomial trend analysis [43]. No significant linear or larger order trend was found (F_(1,17)_ = 2.644, *P* = 0.122). A likelihood ratio analysis (see [44] for details) also reflected the absence of ratio effect (λ = 3.67).

Comparable differences were observed in accuracy. Accuracy was not affected by numerical ratio (*F*_(4,68)_ = 0.572, *P* = 0.684), continuous variables did not affect the performance (*F*_(1,17)_ = 1.285, *P* = 0.273) and the interaction was not significant (*F*_(4,68)_ = 0.383, *P* = 0.820). No significant trend was found (F_(1,17)_ = 0.378, *P* = 0.547). A likelihood ratio analysis also reflected the absence of ratio effect (λ = 1.15).

#### Large numbers

In the large quantity range the reaction time increased with decreasing numerical ratio (*F*_(4,68)_ = 31.889, *P*<0.001), while the continuous variable factor was not significant (*F*_(1,17)_ = 2.880, *P* = 0.108; interaction *F*_(4,68)_ = 2.469, *P* = 0.053, Fig. 3). There was a significant trend (linear trend: F_(1,17)_ = 57.302, *P*<0.001; quadratic trend: F_(1,17)_ = 8.250, *P* = 0.011).

Similarly, accuracy decreased with decreasing numerical ratio (*F*_(4,68)_ = 8.564, *P*<0.001), control of the continuous variables did not affect performance (*F*_(1,17)_ = 0.205, *P* = 0.657) and no interaction was found (*F*_(4,68)_ = 0.359, *P* = 0.837). There was a significant trend (linear trend: F_(1,17)_ = 24.348, *P*<0.001; quadratic trend: F_(1,17)_ = 8.303, *P* = 0.010).

On the whole, accuracy did not differ between the small number range and the large number range (t_(17)_ = 1.877, *P* = 0.078). However, for the ratio of 0.75 the participants were significantly more accurate in the small number range than the large number range (t_(17)_ = 2.197, *P* = 0.042).

### Fish experiment

Data were analysed separately for small number and large number range with a two (experience: juveniles/adults) by five (numerical ratio: 0.25/0.33/0.50/0.67/0.75) between-subject ANOVA.

#### Small numbers

**Figure 4:**
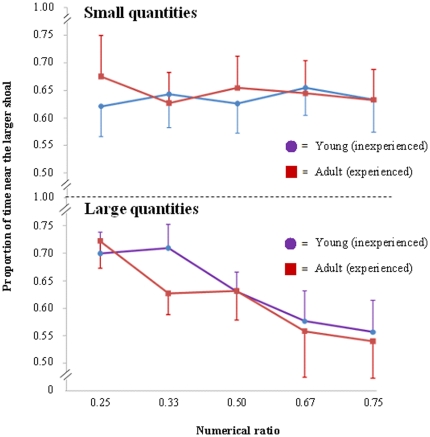
The results of the fish experiment. The proportion of time spent near the larger shoal is plotted against the numerical ratio of the contrasts for both large and small number (1–4) ranges. The performance of the fish (both experienced and inexperienced fish) adhered to the same patterns as for humans in the two numerical ranges, with ratio sensitivity only being shown in the large number range. Bars represent the standard error.

The proportion of time spent near the larger shoal was not influenced by either numerical ratio (F_(4,169)_ = 0.047, *P* = 0.996) or experience (F_(1,169)_ = 0.030, *P* = 0.864), and the interaction was not significant (F_(4,169)_ = 0.242, *P* = 0.914, Fig. 4). No significant trend was found (F_(4, 169)_ = 0.045, *P* = 0.876). Likelihood ratio analysis also reflected the absence of ratio effect (λ = 3.68).

#### Large numbers

In the large number range, the proportion of time spent near the larger shoal was influenced by numerical ratio (F_(4,169)_ = 3.190, *P* = 0.015) but not by experience (F_(1,169)_ = 0.300, *P* = 0.585; interaction: F_(4,169)_ = 0.341, *P* = 0.850, Fig. 4). There was a significant linear trend (F_(4, 169)_ = 3.312, *P*<0.001).

Within the small number range, preference for the larger group was statistically significant for all ratios (0.25: t_(33)_ = 3.265, *P* = 0.003; 0.33: t_(33)_ = 3.260, *P* = 0.003; 0.50: t_(33)_ = 3.537, *P*<0.001; 0.67: t_(33)_ = 4.003, *P*<0.001; 0.75: t_(33)_ = 3.230, *P* = 0.003); in the large number range, the fish spent significantly more time near the larger shoal for ratios 0.25 (t_(33)_ = 6.922, *P*<0.001), 0.33 (t_(33)_ = 5.788, *P*<0.001) and 0.50 (t_(33)_ = 4.466, *P*<0.001), but not for ratios 0.67 (t_(33)_ = 1.481, *P* = 0.148) or 0.75 (t_(33)_ = 1.159, *P* = 0.255).

No single ratio significantly differed between the two ranges (two sample t-test, df = 66 all *P*>0.169). Yet all comparisons had a very low statistical power (0.052≤x≤0.279) and therefore lack of significance could be due to type II error. Sample size calculation indicates that, for each comparison of our experiment, a minimum of 90 subjects was needed in order to reach a 80% power to detect a 20% difference between treatment groups with a two-sided test and alpha = 0.05 [45].

To test if lack of significance of between-range comparisons was due to inadequate sample size we increased the sample size for one numerical ratio (0.67), by testing 64 additional adult fish, 32 in a small number discrimination (2 vs. 3) and 32 in a large number discrimination (4 vs. 6). The difference between the 2 vs. 3 and the 4 vs. 6 contrasts became statistically significant (independent t-test t_(130)_ = 2.688, *P* = 0.008). As observed with humans, as discrimination became more difficult, the fish tended to be more accurate in the small number range; they could in fact discriminate between two numbers with a 0.67 or 0.75 ratio in the small number range, whereas they required a 2∶1 ratio when tested with large numbers.

## Discussion

Previous studies have reported remarkable similarities in the performance of non-verbal numerical tasks among humans, apes and monkeys, suggesting the existence of the same basic numerical systems among primates [3], [29], [46], [47], [48]. Here we provide evidence of a similar correspondence in numerical abilities between humans and teleost fishes.

When tested in the same numerical tasks, the students and guppies showed almost identical performance patterns. In both species, the ability to discriminate between large numbers (>4) was approximate and strongly dependent on the ratio between the numerosities. In contrast, in both fish and students, discrimination in the small number range was not dependent on ratio and discriminating 3 from 4 was as easy as discriminating 1 from 4. Likelihood ratio analyses indicate that the lack of ratio effect is 3.68 times more likely than the alternative hypothesis in fish and 3.67 times in students. As a consequence, the discrimination of larger numerical ratios, 0.67 and 0.75, is easy in the small number range for both species, but becomes relatively more difficult (among students) or even impossible (among fish) when confronted with large numbers.

It is possible that, in fish, the different discriminative ability in the two numerical ranges is the consequence of a different encounter rate with large or small social groups and thus a different familiarity with large and small numbers. However, the observation that juveniles raised in pairs, and therefore with no previous experience in comparing social groups, showed the same pattern as experienced adults rules this possibility out and indicates that numerical systems are probably innate in fish. The observation that some numerical abilities are exhibited by guppies at birth is certainly remarkable and is line with evidence that both chicks and newborns display some rudimentary numerical skills [49], [50]. This reinforces the proposition by some authors of a core knowledge of number, a system of innate numerical representation shared among the different non-human species [3], [51].

Since numerosity normally co-varies with other physical attributes such as the total area occupied by objects, one may argue that in the fish experiment subjects were using these cues instead of numerical information to solve the task. This possibility was not checked in the present study. However in two previous studies we showed that guppies and mosquitofish can discriminate between two schools of fish using the numerosity information only, both within the small number range or outside it [37], [52] and that mosquitofish can discriminate between sets of geometric figures in both numerical ranges even after all continuous variables were controlled for. In particular a recent study using a training procedure has shown no difference in the learning rate between fish trained to use numerical information only and fish trained to use continuous quantities only, suggesting that the number per se is not more cognitively demanding than continuous quantities [53]. On the other hand students showed a very similar performance whether continuous variables were controlled or not, thus making the comparisons fully legitimate.

One can argue that the guppy and student experiments differ in many respects. In particular students were tested in a sequential presentation, whereas fish saw the simultaneous presentation of the numerosity pairs. However in this respect the two experiments may differ less than may appear at first glance. In our test situation the fish could never see the two stimuli binocularly simultaneously. It was possible for a subject to observe both shoals only when swimming perpendicularly to the main axis, which occurred very rarely during a test. Moreover, in this position, each stimulus was seen with a different eye; in this situation, as a consequence of the lateralization of social recognition [54], [55] and reduced inter-hemispheric transfer of information [56], [57], fish cannot be expected to guess the larger group.

The difference in ratio-dependence suggests the existence in fish, as in humans, of two distinct non-verbal mechanisms of numerical representation, one for numbers 1–4 and one for large quantities. Yet the hypothesis that a precise object-file mechanism does underlie small number discrimination also predicts higher performance in the small number range, a finding not evident in our study. However, the lack of a statistical difference between ranges observed in fish experiment may be due to the limit of the procedure adopted. Previous studies using this procedure have shown that accuracy in selecting the larger shoal never exceed 70% even with very easy numerical ratios (guppies [37], [58], mosquitofish [22], topminnows [59], angelfish [60]). Due to large measurement variance combined with small sample size in each numerical contrast, these statistical tests suffered from low power and hence lack of significance may be attributable to a great probability of making a type II error. Sample size calculation indicates that in our experiment a threefold sample size was needed to obtain an adequate statistical power when two treatments had to be compared. As confirmation, a statistical difference emerged between the large and small number range after we increased the sample size in the 0.67 ratio. Regarding this latter evidence, previous work has reported that macaques, mosquitofish, chicks and bees could distinguish two from three items but failed with the same ratio in the large number range [22], [29], [33], [34], [61], even though none of these studies could provide a statistical test to support a difference in performance between the two ranges. Our result highlights the possibility that, as in other research fields [62], [63], [64], many studies are underpowered to detect statistical differences among subgroups.

The results reported here differ from those found in a very recent study on angelfish which discriminated 2 vs. 3 but not 3 vs. 4 fish [60]. Since both guppies and mosquitofish appear to discriminate 3 from 4 fish [22], [37] there might be taxonomic or ecological differences in the numerical abilities among fish species. Other explanations are however possible. Since poeciliids do not form tight shoals, during the experiment, all stimulus fish of one group are usually simultaneously visible to the subject. Angelfish, by contrast, are characterized by a wide lateral body surface and tend to form coherent and synchronized schools; thus, as the number of individuals increases some individuals could often block the sight of other shoalmates, making it difficult for a subject to accurately estimate group numerosity.

Some recent studies found that accuracy was affected by numerical ratio for both small and large numbers leading some researchers to question the existence of two separate numerical systems [16], [65]. It is unclear why some studies find that performance in the range 1–4 is independent of ratio and some do not. A possible explanation for this literature inconsistency, recently proposed by some authors (e.g. [26], [66], [67]), is that small quantities may be represented by both analog magnitude and object-file mechanisms, and that recruitment of one or the other system may depend on context and previous experience. In line with this hypothesis, a recent study found that, unlike controls, a sample of participants with an expertise that requires years of training in estimation of magnitudes showed the typical signature of the analog magnitude system, ratio effect, in the small number range too [Agrillo and Piffer, unpublished].

However that may be, the lack of a ratio effect in the 1–4 numerical range does not necessarily entail the existence of a separate system. As pointed out by Gallistel and Gelman [9], the difference in ratio effect between large and small numbers could occur because there is so little error in the analog magnitude representations of 1, 2, 3, and 4 that they are highly distinguishable from one another, and thus coarse behavioral measures of the underlying processes (as they often are with both human and non-human experiments), fail to evidence a ratio dependence when in fact such a relationship may exist.

Our results do not help to clarify this issue, being compatible with both hypotheses. However, even assuming the existence of a single system of analog magnitude with a different ratio sensitivity in the range 1–4 and beyond it, the similarity in human and guppy experiments in the steepness of the slope in both ranges is again strongly suggestive of similar systems of numerical representation.

Other studies have provided evidence to support strong similarities between teleosts and primates. Swordtails, mosquitofish, angelfish and climbing perch appear to adhere to Weber's Law when discriminating between two large quantities [22], [23], [38], [68] and mosquitofish trained to discriminate between large sets of geometric figures were found to be equally efficient in discriminating 4 vs. 8 items or 100 vs. 200 items, exactly like the college students tested with the same stimuli [36]. Naturally, as with most comparative data, it is always possible that a strong similarity in cognitive abilities is the product of convergent evolution and that similar performance reflect very dissimilar underlying mechanisms.

It might seem surprising to discover similar numerical abilities in humans and in guppies, especially when considering that the brain size of a guppy is less than a thousandth of that of primates. However, it is clear from recent literature that the cognitive abilities of fish have been greatly underestimated and that teleosts are capable of complex behaviors such as individual recognition, transmission of cultural traditions, cooperation, copying behavior and deception, which have traditionally been associated with the evolution of large cortical areas in mammals and birds [69], [70]. On the other hand, it is also possible that a cognitive function such as numerical discrimination, which is apparently complex, may actually be based on relatively simple neural circuits, as suggested by a recent neural network study [71].

On a more general note, the evolution of numerical abilities in animal species is still a largely unexplored field and future research is needed to understand the origin of the quantitative systems shown by vertebrates. If numerical abilities have evolved many times independently in different taxa it would be challenging to understand which selective constraints have shaped them in a converging fashion. On the other hand, the results of this comparative study admits the possibility of common mechanisms between primates and basal vertebrates, suggesting that the evolutionary emergence of numerical abilities may be very ancient, possibly dating back to before the teleost-tetrapod divergence.
